# The *Drosophila melanogaster* Mutants *ap^blot^* and *ap^Xasta^* Affect an Essential *apterous* Wing Enhancer

**DOI:** 10.1534/g3.115.017707

**Published:** 2015-04-02

**Authors:** Dimitri Bieli, Oguz Kanca, Daryl Gohl, Alexandru Denes, Paul Schedl, Markus Affolter, Martin Müller

**Affiliations:** * Biozentrum, University of Basel, 4056 Basel, Switzerland; † Department of Molecular Biology, Princeton University, New Jersey 08540

**Keywords:** *Drosophila*, *apterous*, compartment, boundary

## Abstract

The selector gene *apterous* (*ap*) plays a key role during the development of the *Drosophila melanogaster* wing because it governs the establishment of the dorsal-ventral (D-V) compartment boundary. The D-V compartment boundary is known to serve as an important signaling center that is essential for the growth of the wing. The role of Ap and its downstream effectors have been studied extensively. However, very little is known about the transcriptional regulation of *ap* during wing disc development. In this study, we present a first characterization of an essential wing-specific *ap* enhancer. First, we defined an 874-bp fragment about 10 kb upstream of the *ap* transcription start that faithfully recapitulates the expression pattern of *ap* in the wing imaginal disc. Analysis of deletions in the *ap* locus covering this element demonstrated that it is essential for proper regulation of *ap* and formation of the wing. Moreover, we showed that the mutations *ap^blot^* and *ap^Xasta^* directly affect the integrity of this enhancer, leading to characteristic wing phenotypes. Furthermore, we engineered an *in situ* rescue system at the endogenous *ap* gene locus, allowing us to investigate the role of enhancer fragments in their native environment. Using this system, we were able to demonstrate that the essential wing enhancer alone is not sufficient for normal wing development. The *in situ* rescue system will allow us to characterize the *ap* regulatory sequences in great detail at the endogenous locus.

The body wall and appendages of the adult fly are generated by specialized clusters of primordial cells in *Drosophila* larvae called imaginal discs. The patterning of cells in imaginal discs is initiated by establishing cell lineage boundaries, called compartments (Garcia-Bellido *et al.* 1973; Dahmann and Basler 1999). In the case of the wing imaginal disc, the tissue is subdivided into four different compartments, anterior (A) and posterior (P) as well as dorsal (D) and ventral (V). The A−P compartment is established during the process of segmentation in the embryo. The subdivision into dorsal and ventral compartments takes place later in development during the larval stages when the wing tissue is growing extensively (Wieschaus and Gehring 1976; Lawrence and Morata 1977; Cohen *et al.* 1992; Williams *et al.* 1993; Diaz-Benjumea and Cohen 1993). Short-range signaling events between the A−P or D−V compartments specify cells close to the compartment boundaries. These cells, also called organizer, play an important role in patterning the surrounding tissue by secreting long-range signaling molecules, also referred to as morphogens (Struhl and Basler 1993; Diaz-Benjumea and Cohen 1995; Neumann and Cohen 1997; Affolter and Basler 2007).

Compartment specificity is conferred by the cell-autonomous activity of a special class of transcription factors, called selector genes. Selector genes regulate genes important for proper differentiation and genes that control cell−cell interactions at the compartment boundary. *apterous* (*ap*), which is expressed in the dorsal compartment of the wing disc, has been shown to act as a selector gene subdividing the wing disc into a D and a V portion (Cohen *et al.* 1992; Diaz-Benjumea and Cohen 1993; Williams *et al.* 1994; Blair *et al.* 1994). Different *ap* alleles can lead to a wide range of wing phenotypes (Stevens and Bryant 1985). The most striking morphological defect in strong *ap* alleles is the complete lack of wing and haltere structures (Butterworth and King 1965). Because *ap* is not essential for the progression through larval and pupal stages, the investigation of adult *ap* mutant wing phenotypes is possible.

The target genes of Ap and their downstream functions in the patterning of the wing disc are relatively well understood. The activity of Ap initiates a bidirectional Notch signaling cascade at the D−V compartment boundary, which subsequently induces the expression of *wingless* (*wg*) in a stripe along the compartment boundary (Diaz-Benjumea and Cohen 1993; Williams *et al.* 1994; Irvine and Wieschaus 1994; Rulifson and Blair 1995; Kim *et al.* 1995; Couso *et al.* 1995). Wg, a ligand of the Wnt family, is responsible for the growth of the wing pouch and patterning along the D−V-axis, although its mode of action as a classical morphogen currently is questioned (Neumann and Cohen 1997; Alexandre *et al.* 2014).

Despite the rather detailed knowledge about the functions of Ap in wing disc development, our knowledge of the mechanisms regulating *ap* expression is still limited. It has been shown that activation of the epidermal growth factor receptor by its ligand Vein is necessary and sufficient to activate the expression of *ap* in the dorsal compartment of the wing disc (Zecca and Struhl 2002a,b). Moreover, early ventral *wg* expression has been shown to restrict the expression of *ap* to the dorsal portion of the developing wing disc (Williams *et al.* 1994).

To identify the wing disc-specific *cis*-regulatory elements of *ap*, we used several genetic approaches. First, a classical *LacZ* enhancer reporter study was performed. Second, deletions with defined breakpoints in the *ap* genomic locus were generated. Third, we have characterized two classical *ap* alleles, *ap^blot^* and *ap^Xasta^* (*ap^Xa^*), at the molecular level and have associated their respective molecular alterations to the minimal wing enhancer. Finally, we engineered a *ΦC31*-integrase-dependent *in situ* rescue system, which enabled us to dissect the role of these *cis*-regulatory elements in their native environment.

Using these assays, we have defined an essential, but not sufficient, minimal 874-bp *ap* wing enhancer fragment that drives reporter gene expression in the dorsal compartment of the wing imaginal disc.

## Material and Methods

### Fly stocks and methods

Flies were grown on standard cornmeal agar at 25°, unless otherwise stated. *ap^e01573^* (*PBac{RB}e01573)*, *ap^f08090^* (*PBac{WH}f08090*), *ap^f00451^* (*PBac{WH}f00451*), and *ap^f00878^* (*PBac{WH}f00878*) were purchased from the Exelixis stock collection at Harvard Medical School. *Df(2R)nap1* (BL#1006), *ap^blot^* (BL#4190), w*; *T(2;3)ap^Xa^*, *ap^Xa^/CyO*; *TM3*, *Sb^1^* (BL#2475), *P{hsFLP}12*, y^1^ w* (BL#1929), *Df(2R)BSC696* (BL# 26548), w*; *P{10XUAS-IVS-mCD8*::*GFP}attP40* (BL#32186), *Df(3R)Exel6176* (BL#7655), *TM3*, *ry^RK^ Sb^1^ Ser^1^ P{Δ2-3}99B* (BL#1808), *Bx-Gal4* (*w^1118^ P{GawB}Bx^MS1096^*, BL#8860), y^1^ w*; Mi*{y[+mDint2]=MIC}MI00964* (BL#34133), y^1^ w*; Mi*{y[+mDint2]=MIC}MI02330/SM6a* (BL#33205), *y^1^ w^67c23^*; *P{EPgy2}EY03046* (BL#15619), *ptc-Gal4* (*P{GawB}ptc^559.1^*; BL#2017) were all obtained from the Bloomington Stock Center. *fng-Gal4* (y* w*; *P{w^+mW.hs^ = GawB}NP5399 / TM6*, *P{w^-^=UAS-lacZ.UW23-1}UW23-1*, DGCR#104990) was obtained from Kyoto Drosophila Research Center. *Dad4-Gal4* was established in our laboratory as described in the sections to follow. *actin-Gal4* (*y w^1118^* ; *P{actin5c*::*Gal4*, *w^-^}/CyO*) and *GMR-Gal4* (*w^1118^* ; *P{GMR*::*Gal4*, *w^-^}/CyO*) were obtained from Steven Henikoff (Ahmad and Henikoff 2001). *salE-Gal4* was obtained from the Basler lab via Fisun Hamarotoglu (Mosimann *et al.* 2006). *dpp-Gal4* is described in Staehling-Hampton *et al.* (1994). *UAS-ap* was obtained from Marco Milán (Milán and Cohen 1999). *y w M{vas-int.Dm}zh-2A*, a stock producing *ΦC31*-integrase under the control of the *vasa* promoter, and insertion platform *M{3xP3-RFP.attP}zh-86Fb* were obtained from Johannes Bischof (Bischof *et al.* 2007). *ap^41F^/T(2;3)ap^Xa^* was obtained from John B. Thomas. 

According to our genetic and molecular analysis, *ap^41F^* should not be listed as an allele of *ap*. First, and contrary to a previous report (Bourgouin *et al.* 1992), hemizygous *ap^41F^* flies have normal wings and halters. Second, although molecular analysis confirmed the presence of a *P*-element insertion just proximal to *vulcan* on the *ap^41F^* chromosome, polymerase chain reaction (PCR), and sequencing failed to provide evidence for a ∼200-bp deletion within 1.5 kb of the longest *ap* cDNA (D. Bieli and M. Müller, unpublished data). The *GFP* knock-in allele *ap*::*GFP* is described in Caussinus *et al.* (2011) (BL#38423). *ap^MM^* has been described in Gohl *et al.* (2008). It contains an insertion ∼400 bp upstream of the longest *ap* cDNA. *Dad4-GFP* (*P{Dad4*::*EGFPnuc*, *w^+^}*) was obtained from Jorgos Pyrowolakis (Vuilleumier *et al.* 2010). Nuclear enhanced green fluorescence protein (GFP) is expressed under the control of the Dad4 enhancer (Weiss *et al.* 2010). *dad^P1883Δ32^ / TM3*, *Sb* was obtained from Tetsuya Tabata. This deletion covers at least 24 kb downstream of the *Dad^P1883^* insertion, including the complete *Dad* open-reading frame (ORF) and three neighboring genes (*CG3983*, *CG5184*, and *CG3962*; T . Tabata, personal communication; Tsuneizumi *et al.* 1997; Henderson *et al.* 1999). A recombinant between *ap^Xa^* and *P{y[+t7.7] w[+mC]=10XUAS-IVS-mCD8*::*GFP}attP40*, inserted on 2R at 25C6 (Pfeiffer *et al.* 2010), was obtained by meiotic recombination and selection for the dominant *Xasta* and *mini-white* markers. The generation of deficiencies *ap^DG1^*, *ap^DG3^*, *ap^DG8^*, and *ap^DG11^* is described below in section *ΦC31-integrase–mediated transgenesis and generation of deletions*.

Adult wings were dissected and mounted in Hoyer’s. Then, wing preparations were baked at 58° for a few hours. Preparations were allowed to harden at room temperature and flattened by applying a 40-g metal cylinder on the cover slip. Pictures were taken with a Nikon Microphot-FXA microscope with a Sony NEX-5RK digital camera. The notums of adult flies were photographed with a Leica M125 binocular equipped with a Leica DFC420C camera.

### Introduction of *ΦC31*-integrase targets into the *ap* locus by gene conversion at the site of *ap^MM^*

A method known as direct gene conversion has previously been developed to engineer a desired DNA fragment into the genomic site of a *P*-element insertion (Gloor *et al.* 1991; Sipos *et al.* 2007). Upon exposure of a given *P*-element insertion to *P*-element transposase, the transposon is excised and a double strand break is created. It is normally repaired by the cellular machinery using the homologous chromosome as a template. However, the repair process may also use an exogenous plasmid containing the desired DNA fragment flanked by homology arms derived from either side of the *P*-element insertion site. Such a gene conversion template plasmid containing homology arms flanking the site of *ap^MM^* insertion, along with *hsp70-GFP* bracketed by a pair of inverted attB sites was constructed and named pLAPGPRA (see Figure 5A). The construction of this plasmid was a multi-step procedure. Details can be obtained upon request. In brief, left (899 bp long) and right (1981 bp long) *ap*-homology arms were amplified by PCR. To minimize sequence polymorphism which could decrease the efficiency of gene conversion, *ap^MM^* genomic DNA (gDNA; isolated as described in Ashburner 1989) was used as the template for PCR. As primers we used apLA-R, apLA-FNotI, apRA-F, and apRA-R (for primer sequences, see Supporting Information, Table S1). Inserts in the proper orientation for subsequent cloning were identified using diagnostic digests and sequencing.

pLAPGPRA was injected (650 ng/μL) along with pTurbo (250 ng/μlL) into embryos derived from a cross of *y w*; *ap^DG3^{w^+^}/+*; *TM3*, *Sb Δ2-3/+* males with *y w*; *ap^MM^{y^+^}* ; *+* virgins. Surviving injectees were transferred to fresh vials and carefully tended at 18°. Among the hatching adults, males and virgins representing the two desired genotypes (*ap^DG3^{w^+^}*/*ap^MM^{y^+^}* and *ap^DG3^{w^+^}*/*ap^MM^{y^+^}*; *TM3*, *Sb Δ2-3/+*) were selected for further work. *ap^DG3^*/*ap^MM^* flies have normal wings and halteres. The *ap^DG3^* chromosome was included because it lacks the DNA corresponding to the homology arms of pLAPGPRA and hence cannot serve as a template for double strand gap repair. A total of 72 fertile crosses involving virgins (in pairs) or single males mated with *y w*; *al b c sp/SM6a* flies could be set up. Originally, it was intended to screen the larval progeny of these crosses for GFP expression. Unfortunately, this elegant approach failed in practice. Therefore, the progeny was screened for *y^−^ w^−^* males. This phenotype indicates loss of the *y* marker and therefore most likely also of *ap^MM^* and was, in the absence of the positive GFP selection, the only selectable marker to identify putative conversion candidates. A total of 105 *y^−^ w^−^* males were selected from 32 (out of 72) crosses yielding such males. Balanced lines of potential gene conversion events were established and screened for GFP fluorescence in larval wing discs. Five candidate gene conversion lines with weak GFP expression in wing imaginal discs in an *ap*-like pattern were obtained from two independent dysgenic crosses (isolation numbers: c1.4a, c1.4b, c1.4d, and c1.4e; c1.13a).

To confirm that the five GFP-positive candidate gene conversion lines had the attP-flanked *GFP* construct integrated in the *ap* locus, gDNA was isolated and analyzed by PCR. PCR products were obtained for all five candidates between a primer (SV40out55) within the SV40 trailer sequence (just downstream of *GFP*) and a primer (apLOF2) in the *ap* gene outside of the left homology arm. Sequencing of all five lines confirmed the integrity of the *ap* promoter region and the presence of the *ap* proximal attP site (data not shown).

On the distal side, PCRs using primers in the *hsp70* promoter (just upstream *GFP*) and several primers outside of the right homology arm initially failed to produce products (data not shown). Later, by use of one of the lines obtained by RMCE (see below), the integrity of the junction between the template plasmid and the right homology arm could be verified by PCR and sequencing using the Mcp-dir-y and ap-dir-3 as primers. Mcp-dir-y primes toward the end of the *mini-yellow* gene present on our Recombination-Mediated Cassette Exchange (RMCE) insertion cassette. We also tested whether the junction between the right homology arm and the flanking *ap* sequence is intact by PCR using a primer near the end of the right homology arm (apRAendF), and a primer in the flanking *ap* sequence (apROR2). A product of the expected size was observed, indicating that the junction is intact.

*ap^c1.4a^*, *ap^c1.4b^*, *ap^c1.4d^*, *ap^c1.4e^*, and *ap^c1.13a^* homozygotes all have wild-type wings, indicating that the function of the *ap* wing enhancer and promoter were not disrupted by the gene conversion event. Gene conversion events *ap^c1.4a^*, *ap^c1.4b^*, *ap^c1.4d^*, *ap^c1.4e^* also have a rough eye phenotype when homozygous, but not over *ap^DG3^*. The rough eye phenotype can be separated from the *ap* locus by meiotic recombination. Finally, only *ap^c1.4b^* was chosen for further work. One of its applications is the targeted insertion of exogenous DNA into the *ap* locus by RMCE (Bateman *et al.* 2006).

### *ΦC31*-integrase−mediated transgenesis and generation of deletions

Constructs for *ΦC31*-integrase−mediated transgenesis were generated based on plasmid piB-LLFY(BI) [details about the construction of piB-LLFY(BI) can be acquired upon request]. As required for RMCE, it contains two inverted attB sites. Separating them are the following three genetic components: (1) two LoxP sites in direct orientation with a multiple cloning site in between them; (2) the LoxP cassette is followed by a single FRT site; (3) the *mini-yellow* transformation marker completes piB-LLFY(BI). *mini-yellow* refers to a *yellow* reporter gene lacking all of its characterized tissue specific enhancers. It consists of the *yellow* cDNA fused to ∼330 bp of 5′ genomic DNA, including the yellow promoter and extending up to a *Kpn*I restriction site. The *mini-yellow* fragment was isolated from plasmid C4yellow (referred to as Dint in Geyer and Corces 1987; Gohl *et al.* 2008). In the context of the *ap* gene and in a *y* background, *mini-yellow* activity always manifests itself in phenotypically yellow^+^ wings. Depending on the transgene and orientation of insert, thoracic bristles may also acquire yellow^+^ pigmentation (D. Gohl and M. Müller, unpublished data). 

**Figure 1:**
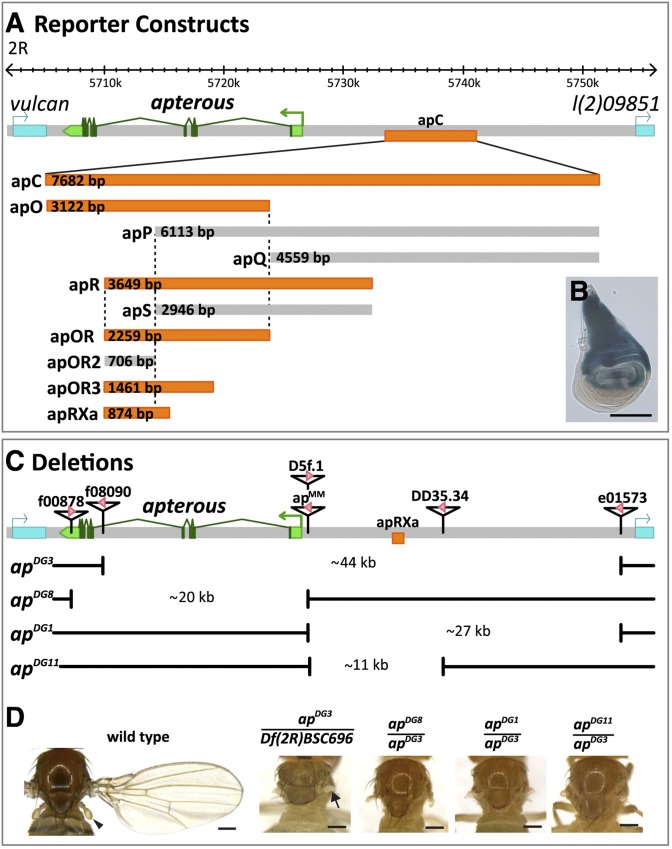
*LacZ* reporter assay and deletion analysis at the *apterous* locus. (A) Diagrammatic representation of the *ap* locus. As drawn at the top of the panel, it extends over roughly 50 kb. Its transcribed part is shown in green. *ap* is flanked by two genes indicated in blue: *vulcan* on the proximal and *l(2)09851* on its distal side. Arrows above the genomic interval specify the direction of transcription of the three genes. Fragment apC, indicated in orange, has been reported to drive reporter expression in the dorsal compartment of the pouch, the hinge and the notum of the wing imaginal disc, where *ap* is normally expressed. Below, the relative positions and dimensions of nine fragments tested with our LacZ reporter assay are depicted. Fragments colored in orange (apO, apR, apOR, apOR3, and apRXa) elicit the same expression pattern as apC. Fragments depicted in gray (apP, apQ, apS, apOR2) do not drive reporter gene expression in the wing disc. (B) X-Gal staining in the wing disc of an *apC-LacZ* transgenic fly. Scale bar: 100 µm. (C) Deletions generated at the endogenous *ap* locus with FRT-containing inserts. At the top of the panel, triangles along the *ap* locus indicate the position of six different inserts. Pink arrowheads within them mark the orientation of the FRT sites according to the definition of Thibault *et al.* (2004). The location of the apRXa fragment is shown in orange. *ap^DG3^* deletes approximately 44 kb between inserts *ap^f08090^* to *ap^e01573^*, thereby removing most of *ap* ORF and upstream sequences. *ap^DG8^* is a 20-kb deficiency that deletes the complete *ap* ORF from *ap^f00878^* to *ap^D5f.1^*. *ap^DG1^* removes the complete intergenic spacer between *ap^MM^* to *ap^e01573^*. *ap^DG11^* deletes an 11-kb fragment from *ap^MM^* to *ap^DD35.34^*. Note that *ap^D5f.1^* and *ap^MM^* have exactly the same insertion site. (D) Notum pictures of a wild-type fly and *trans*-heterozygous *ap* mutants. In the wild type, the wing and the haltere (arrowhead) are well formed and clearly visible. *Df(2R)BSC696* is a large deletion at the base of 2R, deleting approximately 360 kb, including the whole *ap* locus. When *Df(2R)BSC696* is crossed to *ap^DG3^* all wing and haltere structures are lost. Only small stumps of amorphic tissue remain at the actual attachment site of the wing (see arrow).Very similar phenotypes are observed in *ap^DG8^*/*ap^DG3^*, *ap^DG1^*/*ap^DG3^* and *ap^DG11^*/*ap^DG3^* flies. Scale bar: 25 µm.

Constructs were introduced into the *ap* locus by RMCE into two docking sites, *Mi{y[+mDint2]=MIC}MI02330* (Venken *et al.* 2011) and *ap^c1.4b^*. DNA was injected at a concentration of 300 ng/µL in 1× phosphate-buffered saline (PBS) into early embryos of the genotype *y w M{vas-int.Dm}zh-2A*; *MI02330*/*CyO* or *y w M{vas-int.Dm}zh-2A*; *ap^c1.4b^*/*CyO*. The relevant transgenic lines obtained in this way are *ap^DD35.34^* and *ap^D5f.1^*, respectively. Their position and the orientation of the FRT are depicted in Figure 1C together with four other FRT containing transposon insertions. Five of the six stocks are homozygous and hemizygous viable. Their wings and halteres are of wild-type appearance. This is not the case for *ap^f08090^*. The lethality of this chromosome cannot be reverted by excision of the PBac{WH}, indicating that it is associated with a second site lethal. Rare homozygous revertant escapers as well as frequent hemizygous revertants have normal wings. Therefore, the *PBac{WH}* insert is responsible for the strong phenotype in hemizygous *ap^f08090^* flies. However, this phenotype is not dependent on the gypsy insulator present in *ap^f08090^* because the wing phenotype is not suppressed in a *su(Hw)^−^* background (M. Müller, unpublished data).

We have noted that in the *Drosophila* literature, two divergent definitions for FRT orientation are in use! In this study, FRT orientation is indicated according to Thibault *et al.* (2004).

In *Drosophila*, the production of deletions by Flipase-catalyzed recombination between two FRT sites either *in cis* or *in trans* has enabled the community to obtain a huge collection of tailor-made deficiencies (Golic and Golic 1996; Ryder *et al.* 2007). We have previously applied this technology to generate a ∼27-kb deletion named *Df(2R)ap^DG^* between two FRT sites in *ap^MM^* and *ap^e01573^* (Gohl *et al.* 2008). Note that in this study, *Df(2R)ap^DG^* is referred to as *ap^DG1^*. Applying analogous genetic crossing schemes, we have generated three further deletions:

#### Df(2R)ap^DG3^:

An ∼44-kb deletion between two FRT sites located in *ap^f08090^* and *ap^e01573^*. It is referred to as *ap^DG3^*. In this deficiency, a large part of the *ap* transcription unit is lost together with ∼27 kb of intergenic DNA separating *ap* from *l(2)09851*. Although a considerable part of the *ap* ORF located proximal to the break in *ap^DG3^* remains in place, genetic observations are consistent with it being a true null allele with respect to *ap* function in wing and haltere tissue. Flanking the new FRT junction are three genetic elements: *gypsy* insulator and *mini-white* (of *ap^f08090^*) and a splice acceptor (of *ap^e01573^*). Over several kilobases, the region of the new fusion is identical to *PBac{RB}e01573* and, hence, no adequate *ap^DG3^*-specific PCR primers could be designed. Thus, four PCR primer pairs distributed evenly over the ∼44-kb interval missing on the *ap^DG3^* chromosome were tested on *w^1^* and on *ap^DG3^/Df(2R)nap1* flies (*Df(2R)nap1* being a cytologically visible deletion also uncovering *ap*). The absence of the corresponding DNA in the latter could unambiguously be demonstrated (data not shown).

#### Df(2R)ap^DG8^{w^+^}:

An ∼20-kb deletion between two FRT sites located in *ap^f00878^* and *ap^D5f.1^*. It is referred to as *ap^DG8^*. It corresponds to a rather clean deletion of the complete *ap* transcription unit. Its phenotypes are indistinguishable from those observed for *ap^DG3^*. Flanking the new FRT junction are two genetic elements: a UAS-inducible promoter (of *ap^f00878^*) and *mini-white* (of *ap^D5f.1^*). It was verified by PCR and sequencing. Aprec-LA-AscI-F and WARIout#1 primers were used for PCR. For sequencing, we used primers apEnhDel-Seq-PBrev and WARIout#2.

#### Df(2R)ap^DG11^, al:

An ∼11-kb deletion between 2 FRT sites in *al ap^MM^ sp* and *ap^DD35.34^*. It is referred to as *ap^DG11^*. Because *ap^DG1^*, homozygous *ap^DG11^* flies have no wings. Both deficiencies share the same proximal break point. Previous transvection studies have suggested that the *ap* promoter immediately proximal to *ap^DG1^* (and hence also of *ap^DG11^*) remains intact (Gohl *et al.* 2008). Flanking the new FRT junction are two genetic elements: a LoxP site (of *ap^MM^*) and *mini-yellow* (of *ap^DD35.34^*). The new junction was verified by sequencing. For PCR amplification of the region, the apMM-200for and yellow5′out primer pair was used. Part of the fragment was sequenced with yellow5′out and Inverseappromfor.

### Generation of a *ΦC31*-integrase based *in situ* rescue system at *ap*

#### Construction of pBSattBattPLoxFRTy:

Two complementary oligos (attBPfor and attBPrev) containing attB and attP sites in tandem were purchased from Sigma-Aldrich. These oligos were annealed and cloned between the *Xho*I and *Kpn*I sites of pBSIIKS. The new plasmid’s name is pBSIIKSattBattP. A *Xho*I-*Cla*I fragment containing LoxP, FRT, and mini-*yellow* was isolated from piB-LLFY(BI) and subcloned into pBSIIKSattBattP, thereby generating the pBSattBattPLoxFRTy vector used for *ΦC31* integrase mediated transgenesis (see Figure 5B). The attB and attP sites on this vector are separated by only 6 bp. It was assumed that therefore the two elements are too close for efficient intramolecular recombination. The fact the two desired insertions (one in each attP site present in *ap^c1.4b^*, see Figure 5B) could be isolated seems to support this assumption.

#### Generation of ap^attPΔEnh^, a platform for insertion of ap enhancer fragments:

pBSattBattPLoxFRTy DNA was injected into *y w M{vas-int.Dm}zh-2A*; *ap^c1.4b^/CyO* embryos. Yellow^+^ marked flies could be isolated and mated. The desired insert orientation could be identified by PCR using the apdown-forN and aptransch_yw_rev primers. A stock with isolation number 6.1 was selected for correct orientation of insert pBSattBattPLoxFRTy. It is referred to as *ap^attBPFRTy2^* (see Figure 5C; the other insert orientation was also isolated and called *ap^attBPFRTy1^*). We wished to further modify this stock by introducing the same ∼27-kb deletion as in *ap^DG1^*. Therefore, *y w*; *ap^attBPFRTy2^* males were mated with *y w hsFlp*; *PBac{RB}e01573* virgins. Progeny was heat-shocked at three subsequent days during its larval development for 1 hr in a 37° water bath. Hatchlings were individually crossed to *y w M{vas-int.Dm}zh-2A* ; *Sp Pin/CyO* flies. A total of 7 of 80 single crosses produced phenotypically yellow^−^ and white^−^ flies, indicating the loss of all DNA between the FRT sites of *ap^attBPFRTy2^* and *PBac{RB}e01573*, including *mini-yellow* and *mini-white*. The newly established deletion was named *ap^attPΔEnh^* (see Figure 4D) and kept as a *y w M{vas-int.Dm}zh-2A* ; *ap^attPΔEnh^/CyO* stock. The deletion was confirmed by PCR and sequencing with the primer pair apEnhDel_seq_dwnst_for and apEnhDel_seq_PB_rev.

The *ap^attPΔEnh^* chromosome contains a single functional attP site ready for *ΦC31*-integrase catalyzed insertion of pEnh-Reentry derived plasmids (see Figure 5D). Insertion events can be further modified by suitably placed LoxP and FRT sites, allowing for the deletion of the *yellow* marker or the enhancer fragment-*yellow^+^* marker cassette, respectively (see Figure 5E).

#### Generation of pEnh-Reentry constructs:

*yellow^+^* coding sequence and body cuticle enhancer were subcloned into pBSIIKS as a *Bgl*II fragment from C4yellow, thereby generating plasmid pBSIIKS-yellow. Please note that the *yellow* wing enhancer is not part of the *Bgl*II fragment! attB and FRT LoxP fragments were cloned by first annealing and phosphorylating oligos attBtop and attBbottom as well as FRTLoxPtop and FRTLoxPbottom followed by three fragment ligation with pBSIIKS-yellow vector cut with *Sac*I and *Xba*I. The resulting plasmid was called pEnh-Reentry and served as the backbone for all constructs described below.

The 27-kb full-length enhancer was recombineered in pEnh-Reentry from BACR45O18 (purchased from the Berkeley *Drosophila* Genome Project). The left homology arm was amplified with PCR with primers containing *Not*I and *Xho*I sites (primer pair: apenhrecLA_Not_for and apenhrecLA_*Xho*I_rev). The right homology arm was amplified with primers containing *Xho*I and *Bgl*II sites (primer pair: apenhrecRA_*Xho*I_for and apenhrecRA_*Bgl*II_rev). Homology arms were cloned in pEnh-Reentry cut with *Not*I/*Bgl*II as 3 fragment ligation. Recombineering was performed according to Thomason *et al.* 2007. In brief, the pEnh-Reentry-homologyarms vector was linearized with *Xho*I and transformed into bacterial strain DY380 (purchased from NCI at Frederick) pretransformed with BAC45O18 (purchased from BDGP), and pre-induced at 42° for 15 min. Recombinants were selected on ampicillin and screened by PCR. The correct recombineering product’s name is pEnh-Reentry-Full-length.

Dad enhancer fragments and apRXa were amplified from *ap^Xa^* gDNA. First, fragments apRXaDadInt2, DadInt52, and Dad4 were cloned into a pBluescript II KS(+) vector, where the *Xba*I site was mutated previously into a *Avr*II site. For apRXaDadInt52, primers apR_AvrII_for and dadint52_*Xma*I_*Spe*I_rev were used. For DadInt52, primers dadint52_*Xma*I_*Spe*I_rev andXa_brkpnt_AvrII_for were used. To clone Dad4, we used the primer pair dad4_AvrII_for and dad4_*Xma*I_*Spe*I_rev. These fragments were combined via the respective *Spe*I or *Avr*II sites to produce apRXaDadInt52Dad4 and DadInt52Dad4 fusion fragments. These were subcloned from pBluescript II KS(+) via *Avr*II and *Xma*I sites into pEnh-Re-entry cut with *Avr*II and *Age*I. apR, apRXa, apP, and apY were amplified from pEnh-Reentry-Full-length plasmid and cloned into pEnh-Re-entry via *Not*I, *Avr*II or *Age*I sites. To clone apR, primers apR_AvrII_for and apR_*Xma*I_*Spe*I_rev were used. For apRXa, primer pair apR_AvrII_for and apRXa_AgeI_rev was used. To amplify apP, primers apP_*Not*I_for and apP_AvrII_rev were used. apY was amplified using primer pair apY_*Not*I_for and apY_AgeI_rev.

All pEnh-Reentry derived constructs were brought into the *ap* locus by *ΦC31*-integrase mediated recombination (see Figure 4, D and E). DNAs were injected at a concentration of 300 ng/µL in 1×PBS into *y w M{vas-int.Dm}zh-2A* ; *ap^attPΔEnh^/CyO* embryos. Transgenic flies were selected with the help of the *yellow^+^* marker and balanced stocks were generated according to standard genetic procedure.

### Generation of LacZ-reporter lines

*ap* regulatory DNA were amplified via PCR from *y^1^ w^67c23^* gDNA with primers containing restriction enzyme sites as overhangs, and subsequently cloned into plasmid pAttBLaZ (Weiss *et al.* 2010) using the respective enzymes. apC was amplified with the primer pair apC_AscI_for and apC_*Bgl*II_rev. The apC fragment was defined by Lundgren *et al.* 1995. The apO fragment was cloned with the primers apC_AscI_for and apO_*Bgl*II_rev. For apP, primers apC_*Bgl*II_rev and apP_AscI_for were used. To clone apQ, primer pair apC_ *Bgl*II_rev and apQ_AscI_for was used. For apR, the primers apR_AscI_for and apR_*Bgl*II_rev were used. apS was cloned with the primers apS_AscI_for and apS_*Bgl*II_rev. apOR was amplified with apR_AscI_for and apO_*Bgl*II_rev. For apOR2, primers apR_AscI_for and apOR2_*Xba*I_rev were used. apOR3 was amplified with apR_AscI_for and apOR3_*Xba*I_rev. apRXa was cloned with apR_AscI_for and apRXa_*Xba*I_rev.

All the reporter transgenes were generated with the *ΦC31*-based integration system using the landing platform *M{3xP3-RFP.attP}zh-86Fb* (Bischof *et al.* 2007).

### Molecular characterization of *ap^blot^*

Complementation crosses with *ap^blot^* over a set of overlapping *ap* deletions mapped the mutation to am ∼11-kb interval upstream of *ap^MM^*. Therefore, a set of PCR primer pairs was designed to screen for a lesion in that region of *ap^blot^* gDNA. *y^1^ w^67c23^* gDNA served as positive control. With one primer pair, a discontinuity could be identified on the *ap^blot^* chromosome. It could be best reconciled with the presence of a larger insertion of DNA of unknown origin. Inverse PCR (iPCR) was subsequently used to obtain sequence information about the ends of the putative insertion. Toward that end, *ap^blot^* gDNA was digested with *Bsa*WI and ligated with T4 Ligase under conditions as previously described (Ochman *et al.* 1988). Primer pairs used for iPCR on the proximal side of the insertion were iPCR_for and iPCR_rev. Primer pairs used for iPCR on the distal side of the insertion were K_for and L_rev. Following this strategy, sequence information could be obtained for both ends of the inserted DNA. Sequence comparison identified them as LTRs of the *blood* retrotransposon (Bingham and Chapman 1986). To verify the insertion, primers out of *blood* 3′ and 5′ LTR (blood3prime and blood5prime, respectively) were used with primers binding in adjacent *ap* regions (iPCR_for and L_rev, respectively). Sequencing was performed by Microsynth AG, Switzerland.

### Molecular characterization of *ap^Xa^*

The dominant *Xasta* allele was originally induced by X-ray mutagenesis in a stock already containing two large inversions on 2R and 3R (Serebrovsky and Dubinin 1930; Waddington 1940; Lewis 1951; Hetherington *et al.* 1968). The new rearrangement was classified as a reciprocal translocation with breakpoints 41F9-41F11;89E8-89F1. Allelism with *ap* was inferred from noncomplementation with known *ap* alleles (Butterworth and King 1965; Stevens and Bryant 1985). Complementation crosses with a set of small overlapping *ap* deletions failed to narrow down the location of *ap^Xa^*. Hence, the whole *ap* locus was screened by overlapping primer pairs. PCR products obtained from amplification of *ap^Xa^/+* and *y^1^ w^67c23^* gDNA were compared. The analysis of these reactions identified a difference close to the insertion break point found in *ap^blot^*. Again, this region was probed by iPCR. *ap^Xa^/+* gDNA was cut with *Nla*III and religated under diluted conditions. For iPCR, the primer pair iPCR_Xa_rev and 19_for was used. Sequencing of the iPCR product revealed that the reciprocal translocation had fused DNA originating from *dad* locus on 3R to *ap*-specific sequences. The fusion was confirmed by PCR and sequencing with 19_for and a primer in the *dad* region (primer dadint52out). The breakpoint associated with *Xasta* in *ap* was found to be identical in the two stocks *ap^41F^/T(2;3)ap^Xa^* and w*; *T(2;3)ap^Xa^*, *ap^Xa^/CyO*; *TM3*, *Sb^1^*.

### Generation of *Dad4-Gal4* fly line

The minimal *hsp70* promoter was amplified from the pUAST vector with the primer pair hsp70_*Xba*I_for and hsp70_*Bam*HI_rev, then cloned into pBluescript II KS(+) via the *Xba*I and *Bam*HI sites. The Dad4 fragment was amplified from gDNA with the primers dad_*Not*I_for and dad_*Nhe*I_rev, followed by the insertion of the fragment into the *Not*I and *Xba*I digested pBS-hsp70 plasmid. Gal4 was amplified from a pCaSpeR4-Gal4 plasmid, obtained from the lab of Konrad Basler, with the primer pair Gal4_*Bgl*II_for and Gal4_*Hin*dIII_rev. The Gal4 fragment was subsequently cloned into the *Bam*HI and *Hin*dIII digested pBS-Dad4-hsp70 plasmid. We amplified the SV40-PA terminator sequence from the pUAST vector, using the primer pair SV40_*Hin*dIII_for and SV40_*Bam*HI_*Apa*I. The SV40_PA was subsequently inserted into the pBS-Dad4-hsp70-Gal4 plasmid using the *Hin*dIII and *Apa*I restriction sites. Finally, the Dad4-hsp70-Gal4-SV40_PA sequence was subcloned into the pCaSpeR4 vector, using the *Not*I and *Bam*HI restriction sites. Transgenic flies were selected in a *y^1^ w^67c23^* background with the help of the *mini-white* marker. The Dad4-Gal4 insert used in this study is linked to the X chromosome.

### X-Gal staining of imaginal discs

Third instar larvae were cut in half, and the anterior part was inverted and subsequently fixed in 1% glutaraldehyde (Fluka) in PBS for 15 min on ice. After fixation, the fixative was removed and the larvae were washed twice with PBST (0.1% Tween 20 in PBS). The tissue was then stained as previously described (Ashburner 1989). Afterward, the imaginal discs were dissected and mounted in 80% glycerol. Discs were analyzed under the Zeiss Axiophot microscope and photographed with a Sony NEX-5RK digital camera.

### *In situ* hybridization

A 1.5-kb fragment from the 3′ end of the *ap* cDNA was amplified from the cDNA clone HL02012 (purchased from DGRC) with primers insitu_*Sac*I_for and insitu_*Kpn*I_rev. The fragment was cloned between *Sac*I and *Kpn*I sites of pBluescript II KS(+) vector. Then, the resulting plasmid was linearized with *Acc*65I and digoxigenin-(DIG)-labeled RNA was produced from T7 promoter according to the manufacturer’s protocol (Roche, Switzerland). *In situ* hybridizations were performed as described in Tautz and Pfeifle (1989). Wing imaginal discs were dissected and mounted in 80% glycerol and photographed under a Nikon Microphot-FXA microscope with a Sony NEX-5RK digital camera.

### Immunostaining

The anterior part of third instar larvae was inverted and fixed with 4% paraformaldehyde in PBS for 25 min at room temperature. Standard protocols were used to perform immunostaining. As primary antibodies, rabbit α-GFP (1:1000; Abcam) and mouse α-Wg (1:120, DSHB, University of Iowa) were used. α-rabbit AlexaFlour488 and α-mouse AlexaFlour568 (Molecular Probes) were used at a 1:750 dilution. Samples were mounted in Vectashield (Vector Laboratories, Inc.). Confocal imaging was performed using a Leica SP5 microscope with a vertical step size of 1 µm. Image processing was done with the ImageJ software.

## Results

### Defining a short wing-specific enhancer element in apC

At *apterous*, four different transcripts starting from three different promoters have been annotated (see www.flybase.org). In this study, the transcription start site for transcripts *ap-RA* and *ap-RC* will be referred to as *ap* TSS.

An ∼8-kb DNA fragment named apC located several kilobases upstream of the *ap* TSS had been shown to drive reporter gene expression in an *ap*-specific pattern in the wing disc (Lundgren *et al.* 1995). We used a *LacZ* reporter assay to analyze the *cis*-regulatory elements in apC in more detail. apC was first sub-divided into four overlapping fragments, apO, apR, apP and apQ (Figure 1A). Of these, only the two promoter proximal fragments, apO and apR were found to drive reporter gene expression in the wing disc. To further pinpoint the wing disc enhancers, we generated five subfragments that span the DNA sequences covered by apO and apR. As shown in Figure 1A, this analysis defines a minimal 874-bp fragment, apRXa. Because apP and apOR2 together cover the minimal apRXa element, but neither showed any expression in the wing disc, key *ap* wing enhancer elements are likely to be on both sides of the breakpoint that divides these two fragments.

To determine whether the wing enhancer element identified in the *LacZ* reporter assay is necessary for the proper regulation of the endogenous *ap* gene, we generated several deletions with defined breakpoints (Figure 1C; for details see the section *Materials and Methods*). The largest of these, *ap^DG3^*, removes almost the entire *ap* locus, from the 4^th^ intron to a site located about 500 bp upstream of the flanking distal gene, *l(2)09851*. Previous observations suggested that *l(2)09851* activity is not affected by the proximity of *ap^DG3^*’s distal break (Gohl *et al.* 2008). As a homozygote or when *in trans* to a large deficiency, *Df(2R)BSC696*, that includes the entire ap locus, *ap^DG3^* flies displayed a complete loss of all wing and haltere structures (Figure 1D). This result suggests that *ap^DG3^* represents an amorphic allele of *ap*, at least with respect to Ap function during wing development. Furthermore, we generated a deletion, called *ap^DG8^*, which removes the whole ORF from the end of the 3′UTR to ∼400 bp upstream of the *ap* TSS. Trans-heterozygous *ap^DG8^*/*ap^DG3^* flies again showed a typical *ap*-null mutant phenotype. Finally, two deficiencies affecting only the 5′ regulatory region were generated, namely *ap^DG1^* and *ap^DG11^*. They share the same proximal break located ∼400 bp upstream of the *ap* TSS. Previous transvection studies suggested that the activity of the *ap* promoter is not affected by this breakpoint (Gohl *et al.* 2008). *ap^DG1^* extends ∼27 kb distally to the same position as *ap^DG3^*. *ap^DG11^* removes only ∼11 kb of upstream DNA, including the whole apC fragment. *ap^DG1^/ap^DG3^* as well as *ap^DG11^/ap^DG3^* flies lacked all wing and haltere structures (Figure 1D). In these two deletions, the minimal wing enhancer element defined by apRXa is removed, suggesting that elements within apRXa are indeed necessary for the regulation of *ap* in the endogenous locus (see Figure 1C).

Although *ap* is expressed in the presumptive notum of the developing wing disc, the phenotypic appearance of the adult dorsal thorax is only mildly affected in flies lacking any *ap* activity (*e.g.*, *ap^DG8^/ap^DG3^* or homozygous *ap^DG8^*). Apart from a few missing macro- and microchaetae in the vicinity of the wing appendage, it appears largely normal (Figure 1D and data not shown). The reduced size of the dorsal thorax and the aberrant bristle pattern in *ap^DG3^/Df(2R)BSC696* flies can probably be attributed to other genetic loci deleted in *Df(2R)BSC696*.

Apart from the dominant *ap^Xa^* allele, lesions in the *ap* gene have been reported as recessive in genetic character. Careful inspection of wings obtained from flies heterozygous for any of the 4 deletions presented in Figure 1C corroborated this fact. However, ∼2% of them had small margin defects, indicating a mild dominance of strong *ap* loss-of-function alleles (data not shown).

### Mutations in the apR region result in wing phenotypes

**Figure 2:**
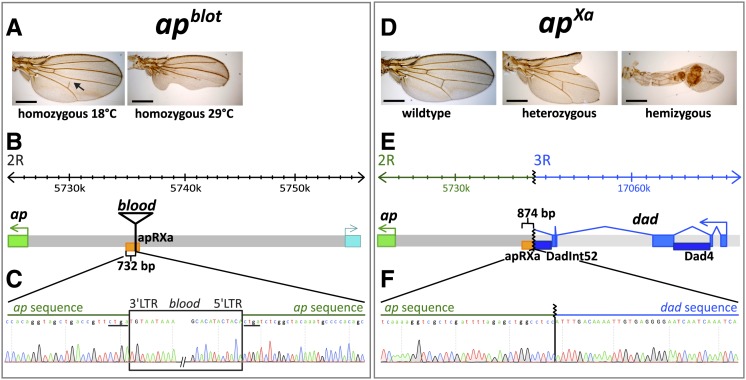
The mutations *ap^blot^* and *ap^Xa^* affect the *ap* wing enhancer region. (A) Temperature-sensitive wing phenotypes obtained for the homozygous *ap^blot^* allele. At 18°, less than 30% of the wings are affected and most of them only show a disruption of the posterior crossvein (arrow). At 29°, ∼70% of the wings have a phenotype. In many of them, the posterior compartment is severely affected. (B) At the top of the panel, the coordinates of the *apterous* locus are indicated. The insertion site of *blood*, a retrotransposable element, within the apRXa wing enhancer is depicted. (C) Sequence data close to the insertion site of the *blood* element in *ap^blot^*. The insertion causes a four bp duplication (CTGA, underlined). Exact coordinates of the 4 bp duplication: 2R:5735176.0.5735179 (Flybase Release FB2014_06). (D) Preparations of wild type and *ap^Xa^* mutant wings. All *ap^Xa^*/+ flies show a dominant phenotype: the distal part of the wing blade is lost and the characteristic mitten phenotype is formed. In hemizygous condition, the wing tissue of *ap^Xa^*/*ap^DG3^* flies forms a short tube-like structure. Margin bristles are absent except for sometimes a few at the tip. All scale bars are 50 µm. (E) Molecular characteristics of the *ap^Xa^* mutation. A reciprocal translocation involving the right arms of the second and third chromosome causes a breakpoint just upstream of the apRXa wing enhancer (indicated in orange) and juxtaposes the *daughters against dpp* (*dad*) locus (indicated in blue) next to the *ap* gene. The dark blue rectangles represent the well-studied *cis*-regulatory elements Dadint52 and Dad4 which are active in the wing disc (Weiss *et al.* 2010). (F) Chromatograph of the *ap^Xa^* sequence across the rearrangement break point. The coordinates of the breakpoints are: 2R:5375319 and 3R:17065902 (Flybase Release FB2014_06).

In the course of investigating the *cis*-regulatory region of *ap*, we identified two classical *ap* alleles, *ap^blot^* and *ap^Xa^*, that map to the apR region. *ap^blot^* was isolated as a spontaneous, hypomorphic mutation that causes notching mostly of the posterior wing margin in homozygous mutant flies, while the anterior wing margin remains largely unaffected (Figure 2A; Butterworth and King 1965; Whittle 1979). To narrow down the genomic site affected by the mutation, intragenic complementation crosses with the aforementioned deletions were analyzed. They showed that *ap^DG11^* was the smallest deletion that failed to complement *ap^blot^*. This observation suggested that *ap^blot^* maps to the ∼11-kb interval defined by *ap^DG11^*. Consequently, this region was screened with a set of overlapping PCR primer pairs. One primer pair did not yield a PCR product and thus identified the site of the putative lesion on the *ap^blot^* chromosome. Using iPCR, we identified the insertion of a retrotransposable element of the *blood* family in the apRXa sequence (Figure 2B, see the section *Materials and Methods* for details). This event caused the typical 4-bp duplication at the insertion site characteristic for *blood* family transposons (Figure 2C; Bingham and Chapman 1986; Wilanowski *et al.* 1995).

Phenotypes caused by *blood* insertions at other loci are sometimes temperature-sensitive (Bingham and Chapman 1986). To test this possibility, we raised homozygous *ap^blot^* flies at different temperatures and scored their wing phenotypes (Table 1). At 18°, only 28% of the wings displayed minor defects. In most of these, the posterior cross vein failed to connect with the 4^th^ wing vein (Figure 2A). At greater temperatures, more severe wing phenotypes were detected with a higher penetrance. At 25° and 29°, 52% and 70%, respectively, of the wings showed extensive notching within the posterior compartment and reduced wing size (Figure 2A).

**Table 1:** Temperature sensitivity of *ap^blot^*

Temperature	Total Wings Scored	Normal Wings	Wings with Phenotypes
18°	294	72%	28%
25°	284	48%	52%
29°	242	30%	70%

**Figure 3:**
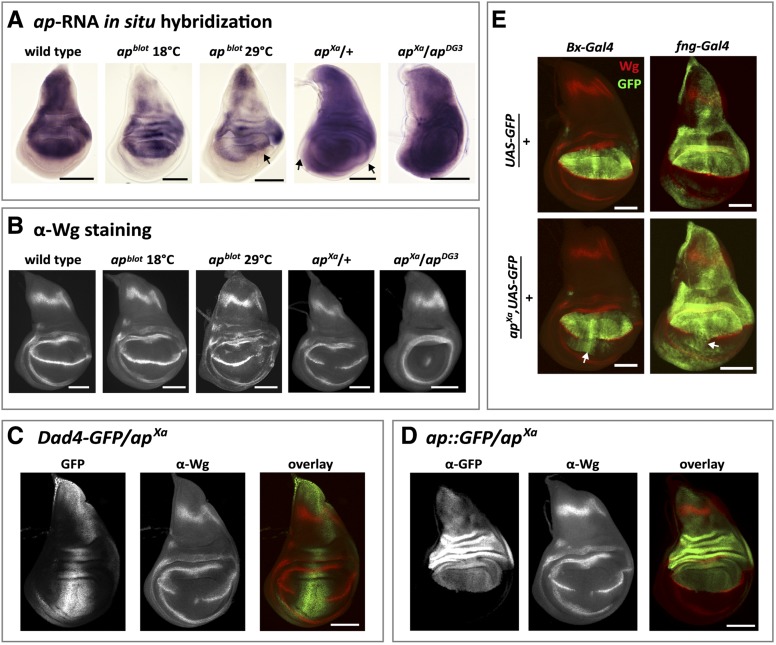
Wing disc phenotypes in *ap^blot^* and *ap^Xa^*. All discs are shown anterior to the left and dorsal side up. (A) *in situ* hybridization against *ap* mRNA in late 3^rd^ instar larval wing discs. In wild type, the dorsal compartment of the wing pouch is filled and outlined by the *ap* transcript. *ap^blot^* discs show reduced *ap* mRNA levels. At 18°, the *ap* expression pattern remains very similar as that in wild type. At 29°, expression of *ap* in the posterior compartment is disturbed and the tissue is deformed (see arrow). In heterozygous *ap^Xa^* discs, ectopic *ap* expression is seen in the ventral part of the wing disc, with the strongest signal in median regions. The black arrows point to the edges of the disc where *ap* transcript is absent. In hemizygous *ap^Xa^*/*ap^DG3^* larvae, a similar pattern is observed. Note the change in shape of the wing disc. (B) α-Wg antibody staining of 3^rd^ instar wing discs. In wild type, a characteristic thin stripe of Wg traverses the wing pouch along the D-V compartment boundary. In *ap^blot^*, Wg expression is normal at 18°C. At 29°C, the Wg stripe is much weaker and less well defined in posterior regions of the wing pouch. In *ap^Xa^*/+ discs, the Wg stripe is interrupted in the median pouch region. In hemizygous *ap^Xa^* discs, the Wg stripe is lost and only a dot of Wg expression in the middle of the pouch is visible. In addition, the size of the pouch is reduced. (C) GFP expression driven by the Dad4 enhancer is detected in the central part of an *ap^Xa^*/+ wing disc. Note that absence of Wg stripe correlates well with higher GFP levels. Therefore, stripe formation is more affected in the anterior than in the posterior compartment. (D) α-GFP and α-Wg antibody staining of an *ap*::*GFP*/*ap^Xa^* wing disc. GFP expression is restricted to the dorsal compartment of the wing pouch. In particular, Ap-GFP fusion protein does not spread ventrally where the Wg stripe is interrupted. This indicates that *dad* enhancers on the *ap^Xasta^* chromosome are unable to activate *ap*::*GFP* located on the homologous chromosome. (E) Expression of *Beadex*- and *fringe-Gal4* enhancer trap lines in wild type and *ap^Xa^*/+ discs. Note that ectopic expression (white arrows) of these two validated Ap targets in the ventral compartment is only detected where the Wg stripe is interrupted. All scale bars are 100 µm.

The dominant *ap^Xa^* allele was generated by X-ray mutagenesis and is associated with a reciprocal translocation between chromosome arms 2R and 3R. The breakpoints were mapped to 41F and 89EF, respectively (Serebrovsky and Dubinin 1930; Waddington 1940; Lewis 1951; Hetherington *et al.* 1968). When heterozygous, *ap^Xa^* flies show the characteristic dominant mitten-shaped wing phenotype, in which the distal tip of the wing is missing leading to a deep notching of the wing blade. In hemizygous *ap^Xa^* flies, only long wing stumps with little or no wing margin and unstructured vein patterns are formed (Figure 2D). The break on 2R has long been known to affect the *ap* locus (Butterworth and King 1965; Stevens and Bryant 1985). However, our attempt to map *ap^Xa^* by intragenic complementation was not successful, suggesting that the lesion in *apterous* prevents this type of genetic analysis (see also Figure 3D). Thus, we screened the entire *ap* locus with overlapping PCR primer pairs. We identified a discontinuity in the apR region and determined the molecular nature of the breakpoint (Figures 2, E and F; for details see the section *Materials and Methods*). It localized right at the edge of the apRXa fragment, 142 bp distal to the insertion site of the *blood* transposon in *ap^blot^*. Only the proximal 874 bp of apR remain associated with the *ap* transcription unit (see Figure 2E). The DNA on the other side of the breakpoint is from the *daughters against dpp* (*dad*) locus located at 89E on 3R. As predicted from the cytological mapping of the rearranged *ap^Xa^* chromosomes, the *dad* locus is inverted compared to its wild-type orientation on 3R (for a comprehensive drawing of the *ap^Xa^* polytene chromosomes, see Hetherington *et al.* 1968). We were not able to determine the breakpoint at the reciprocal site of the translocation. Nevertheless, based on its reciprocal nature, it is conceivable that the *dad* locus is split within its 4^th^ intron and hence destroyed. Because *dad* is expressed in the imaginal wing disc, it is formally possible that the Xasta phenotype is due to the loss of Dad activity. This possibility was addressed by crossing *ap^Xa^* with 2 known *dad* deletions, *Df(3R)Exel6176* and *dad^P1883Δ32^*. The wings of *trans*-heterozygous animals displayed the characteristic mitten phenotype seen in *ap^Xa^* heterozygous flies, suggesting that an amorphic *dad* background does not modify the Xasta phenotype. Hence Dad function is not relevant for the production of the Xasta phenotype (data not shown). This is not unexpected, since *dad* mutants show no visible phenotype in the adult wing (Ogiso *et al.* 2011).

The proximity of *dad* enhancers to the *ap* transcription unit in the *ap^Xa^* chromosome suggests a plausible explanation for the Xasta wing phenotype. Two *cis*-regulatory elements, Dad4 and DadInt52, are located in the *dad* introns (Figure 2E) and are known to drive reporter gene expression in the wing disc in a stripe along the A−P compartment boundary in response to Dpp signaling (Weiss *et al.* 2010). Because *dad* territory encompasses not only the dorsal but also the ventral compartment of the presumptive wing pouch, a likely scenario is that the *ap* promoter responds to these two *dad* enhancers, leading to ectopic Ap expression in the ventral compartment of the pouch.

### Ectopic expression of *ap* in *ap^Xa^* leads to the ectopic expression of Ap target genes

To further characterize the effect of the *ap^blot^* and *ap^Xa^* mutations on wing development, we examined *ap* mRNA and Wingless protein (Wg) expression in 3^rd^ instar larval wing discs (Figure 3, A and B). In wild-type discs, *ap* mRNA is restricted to the dorsal compartment of the wing pouch, the hinge and the notum (Figure 3A). In the pouch, Ap activity is required to direct the expression of Wg in a stripe along the D−V compartment boundary (Figure 3B). This Wg stripe is essential for the proper formation of the wing margin (Couso *et al.* 1994).

The temperature sensitivity of *ap^blot^* was faithfully recapitulated by the expression patterns in 3^rd^ instar wing discs. Although *ap* mRNA levels were reduced at 18° as well as at 29°, an obvious deviation of the *ap* mRNA pattern was only observed at 29° in the posterior compartment of the pouch. This change correlated with a size reduction of the posterior compartment and the appearance of additional tissue folding in this region (arrow in Figure 3A). Consistent with the sharp boundary of the *ap* mRNA expression pattern at 18°, the Wg stripe along the D-V compartment boundary remained unchanged (Figure 3B). In contrast, at 29°, the fuzzy appearance of the *ap* mRNA pattern in the posterior compartment correlated with the disruption of the Wg stripe. In summary, these results are consistent with the adult wing phenotypes and provide an explanation for the abnormalities in the posterior wing margin as well as for the reduced size of the posterior compartment in *ap^blot^* flies raised at elevated temperature.

In *ap^Xa^* heterozygotes, a strong ectopic misexpression of the *ap* transcript was detected in the ventral compartment of the wing disc, with the highest signal along the medial part of the disc (Figure 3A). As a consequence, the Wg stripe was disrupted in the medial region of the wing pouch (Figure 3B). Remarkably, the disruption of the Wg stripe correlated well with the expression domain of the *Dad4-GFP* reporter construct (Figure 3C). Wherever GFP was detected, the expression of Wg was either very low or absent. Wing discs of hemizygous *ap^Xa^*/*ap^DG3^* flies showed strong *ap* expression in the entire pouch region. The characteristic Wg stripe in the wing pouch was lost, leaving behind only a small dot of Wg expression in the middle of the pouch. Moreover, the dimension of the wing pouch was reduced to about half the size of a wild-type pouch.

In *Drosophila*, the somatic pairing of the two homologous chromosomes can lead to a special situation of gene regulation called transvection (Lewis 1954; Sipos *et al.* 1998; Morris *et al.* 1999; Coulthard *et al.* 2005). In this case the regulatory elements of a gene can regulate the expression of its homolog *in trans*. Transvection has been described for many gene loci (for reviews see Wu and Morris 1999; Duncan 2002) including the *ap* locus (Gohl *et al.* 2008). Therefore, we decided to test the transvection ability of *ap^Xa^* by crossing it with ap::GFP. In this combination only the gene *in trans* is labeled with GFP, allowing for the independent detection of the gene product from this chromosome. Trans-heterozygous *ap*::*GFP*/*ap^Xa^* flies displayed no ectopic expression of Ap-GFP in the ventral wing pouch (Figure 3D). This result demonstrates that the misexpression of *ap* is limited to the chromosome affected by the rearrangement.

As a selector gene, *ap* is known to regulate multiple downstream genes (Bronstein *et al.* 2010). We wished to know whether the ectopic Ap expression observed in heterozygous *ap^Xa^* flies was sufficient to induce its targets also in the ventral compartment of the pouch. Toward that end, we analyzed Gal4-enhancer trap lines of two validated Ap target genes, *Beadex* (*Bx*) and *fringe* (*fng*) (Irvine and Wieschaus 1994; Milán *et al.* 2004). Their activity was monitored with the help of a UAS-CD8-GFP transgene. Under wild-type conditions, *Beadex > GFP* expression was detected exclusively in the dorsal wing pouch, whereas *fng > GFP* was observed predominantly in the whole dorsal compartment with weak ventral GFP outside the wing pouch (Figure 3E). When analyzed in a heterozygous *ap^Xa^* background, the expression of both reporters extended to the medial-ventral region of the wing discs (Figure 3E). In summary, the data presented in Figure 3 strongly suggest that in *ap^Xa^*, *ap* is ectopically expressed due to the juxtaposition of *dad* wing enhancer elements and the *ap* promoter. As a consequence, Ap target genes are up-regulated in the ventral compartment of the wing pouch. These molecular events correlate well with the disruption of the Wg stripe in median parts of the wing disc and, finally, the altered adult wing morpholgy.

### Requirements for ectopic wing margin induction

**Figure 4:**
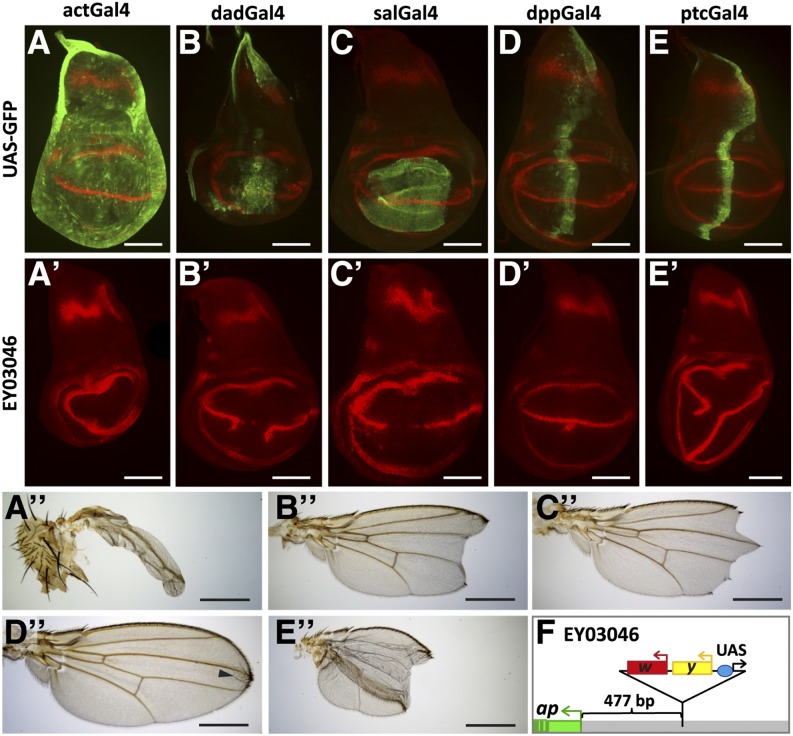
Margin formation in adult wings depends on well-defined On-Off Apterous expression levels during larval development. All discs are shown anterior to the left and dorsal side up. (A−E) 3^rd^ instar imaginal wing discs showing UAS-GFP patterns (in green) elicited by the five Gal4 drivers indicated at the top of the panel. α-Wg antibody staining (in red) outlines the pouch and the position of the D−V compartment boundary. (A´−E´) α-Wg antibody staining. The effect of ectopic Ap production as a consequence of Gal4 > EY03046 on D-V boundary formation is shown. (A´´−E´´) Adult wings as obtained after ectopic Ap expression in (A´´) *actin > EY03046*, (B´´) *Dad4 > EY03046*, (C´´) *salE > EY03046*, (D´´) *dpp > EY03046*, and (E´´) *ptc > EY03046* animals. In (D´´), the arrowhead points to a small lesion near the tip of the wing. Scale bars in (A−F) and (A´−F´) are 100 µm. Scale bars in (A´´−F´´) are 50 µm. (F) Insertion site of *P{EPgy2}EY03046* relative to the *ap* TSS is show*n*. The triangle depicts the structure of the transgene. The red box corresponds to the *mini-white* marker, the yellow box to the *yellow* marker and the blue oval to an array of UAS sites. Arrows specify the transcriptional direction of *mini-white*, *yellow*, and the UAS-driven promoter. *P{EPgy2}* transgenes are intended for regulated expression of genes proximate to the site of the insertion: genes in direct orientation with respect to the UAS-controlled promoter can be conditionally expressed via transgene-derived Gal4 activity (Bellen *et al.* 2004). Note that at *apterous*, the UAS-driven promoter is at a considerable distance from and in opposite orientation to the *apterous* promoter (shown in green). We propose that in *Gal4 > EY03046* flies, Gal4 activates *ap* transcription in much the same way as the eye-specific *GMR-Gal4{w^-^}* driver boosts *mini white* expression in *GMR-Gal4{w^-^}/EY03046* flies. These have red eyes while the eye pigmentation in *EY03046/+* flies is faint yellow (M. Müller, unpublished data). Drawing not to scale.

A puzzling observation is that Ap expression does not induce Wg in the ventral part of the pouch, ultimately leading to extra margin formation in adult wings. One explanation for this finding is that compartment and compartment boundaries must be defined by a clear on-off state of selector gene activity. However, the *dad* gene and its enhancers are regulated by a Dpp concentration gradient (high Dpp in medial parts, low Dpp in lateral parts of the wing disc; for a review see Affolter and Basler 2007). As a consequence, in *ap^Xa^*, Ap is expressed in the ventral part of the wing pouch in response to the *dad* enhancers in a gradient-like manner. Thus, no clear selector gene on-off state between neighboring cells would be generated. In this case, the initiation of the signaling cascade that usually induces the *wg* gene at the compartment boundary fails to be activated.To explore this possibility in more detail, we used the Gal4/UAS-system (brand and perrimon 1993). Preliminary test crosses indicated that upon Gal4 activation, a *UAS-ap* transgene leads to lethality or pleiotropic phenotypes with all Gal4 drivers tested except *dpp-Gal4*. For this reason, we tested an insertion into *ap*, *EY03046*, which contains a UAS-driven promoter located several 100 bp upstream of the *ap* TSS (Figure 4F). In contrast to *Gal4 > UAS-ap* combinations, *Gal4 > EY03046* flies were viable and obvious phenotypes were restricted to the dorsal thoracic appendages. One possible explanation for the difference is that the activation of *EY03046* by Gal4 is reduced or eliminated by the *ap* PRE (Schwartz *et al.* 2006; Tolhuis *et al.* 2006; Oktaba *et al.* 2008; D. Bieli *et al.*, unpublished data) in most tissues outside of the wing disc. To activate *EY03046* expression in the wing pouch, we used the following Gal4 drivers: *actin-Gal4*, *dad4-Gal4*, *salE-Gal4*, *dpp-Gal4*, and *ptc-Gal4*. Their expression domains are depicted in Figure 4, A−E. For our purposes, they can be grouped into three classes: (1) *actin > GFP* is found in all cells of the pouch; (2) *Dad4 > GFP* and *salE > GFP* expression domains are rather broad with a rather ill-defined edge and centered on the A-P axis; and (3) *dpp > GFP* and *ptc > GFP* form a narrow stripe along the A-P axis.

To analyze the effects of ectopic Ap expression, we examined Wg stripe formation along the D-V compartment boundary (see Figure 4, A´−E´) and adult wing morphology (Figure 4, A´´−E´´). Ubiquitous Ap expression in the pouch using *actin*::*Gal4* prevents Wg activation. As a consequence, margin formation in the tiny adult wings was abolished. As expected from the data in Figure 3, Dad4 and *spalt*-mediated Ap expressions led to Xasta phenocopies. Wg stripe formation was abolished in the center of the pouch. Occasionally, small ectopic Wg stripes extended into the ventral compartment in *Dad4 > EY03046* wing discs. Nevertheless, both Gal4 drivers elicited similar moderate Xasta-like phenotypes in adult wings. Finally, although the expression patterns mediated by the *dpp-Gal4* and *ptc-Gal4* drivers were remarkably similar, their phenotypic consequences were dramatically different. *dpp > EY03046* caused the appearance of a faint ectopic Wg patch on the A-P axis in the ventral compartment. A tuft of ectopic bristles was observed on the ventral side at the intersection of the A-P axis and the wing margin in less than 10% of the adult wings (black arrowhead in Figure 4D´´). *ptc > EY03046*, on the other hand, interrupted the Wg stripe in the center of the pouch and frequently induced a well-defined Wg stripe which traversed the whole ventral compartment. A Wg stripe of variable length also is formed in the anterior compartment. Adult wings of this genotype often formed three-dimensional, balloon-like structures with an oval-shaped posterior margin extending from the proximal edge of the wing appendage to its distal end and back to proximal. In addition, anterior and posterior margins were not continuous at the tip of the wing.

These observations corroborate our expectations. First, the presence of Ap in the ventral compartment at sufficiently high levels impedes the activation of the signaling cascade that induces Wg expression along the D−V compartment boundary. Second, an ectopic compartment boundary can only be formed between cells with sharp on-off levels of Ap. This prerequisite is only satisfied by the *ptc-Gal4* driver. In the wing disc, Ptc is expressed in a straight line immediately abutting the posterior compartment where it serves as a receptor for the Hedgehog ligand (Capdevila *et al.* 1994; Alexandre *et al.* 1996). Its anterior limit of expression is more graded and less well defined and ectopic anterior margin in the adult wing can only rarely be observed. The question remains why *dpp > EY03046* is only marginally active in this experiment. It is possible that ectopic Ap levels remain below a certain threshold because the levels of Gal4 do not suffice. Alternatively, the onset of Gal4 activity in this driver line might be delayed. However, very similar observations were made with a *UAS-ap* transgene in place of *EY03046* and also with a different *dpp-Gal4* driver line. In addition, Klein *et al.* have reported a similar phenotype for ectopic Ser expression by *dpp-Gal4* (Klein *et al.* 1998).

### The *in situ* rescue system

**Figure 5:**
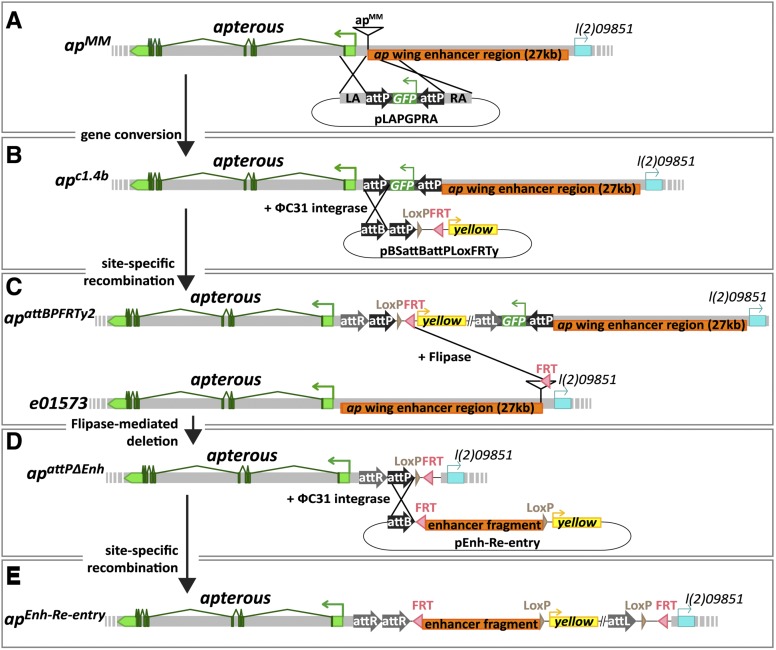
Generation of the *in situ* rescue system at the endogenous *ap* locus. (A−B) Direct gene conversion at *apterous*. P-element insertion *ap^MM^* located ∼400 bp upstream of the *ap* TSS was previously isolated. By mobilization of *ap^MM^* and concomitant injection of plasmid pLAPGPRA, fly line *ap^c1.4b^* could be isolated. It contains two inverted attP sites flanking a GFP reporter. (B−C) *ΦC31*-integrase mediated site-specific recombination. By injection of plasmid pBSattBattPLoxFRTy, new attP, LoxP, and FRT sites were introduced into the *ap* locus. Note that pBSattBattPLoxFRTy can insert in two different attP sites leading to oppositely oriented insertions. *ap^attBPFRTy2^* is the appropriate one for our purpose. (C−D) Flipase-mediated deletion. Trans-heterozygous *ap^attBPFRTy2^*/*ap^e01573^* animals were repeatedly treated with Flipase during larval stages. Among the progeny of these flies, *ap^attPΔEnh^* could be isolated. It lacks the 27kb intergenic spacer but retains a strategically positioned attP site. (D−E) *ap^attPΔEnh^* serves as a platform to reinsert enhancer fragments. These are cloned into pEnh-Reentry. This plasmid is injected into young embryos and integrates into the *ap* locus by *ΦC31*-integrase mediated recombination. Transgenics of the type *ap^Enh-Reentry^* can be isolated thanks to the *yellow* marker. If desired, *yellow* can be removed by Cre-treatment. In addition, the complete insert can be excised by Flipase treatment.

To extend our analysis of the *cis*-regulatory elements directing *ap* expression, we decided to characterize and manipulate possible regulatory sequences directly at the endogenous locus. For this purpose, we engineered an *in situ* rescue system. The establishment of this system was a multistep procedure and is described in detail in the section *Materials and Methods*. A diagrammatic summary is presented in Figure 5. In brief, we deleted the 27-kb intergenic spacer between the *ap* and *l(2)09851* loci and replaced it with an attP site located 400 bp upstream of the *ap* TSS (Figure 5, A−D). This *ap* allele is referred to as *ap^attPΔEnh^* (Figure 5D). The deleted region is identical to that of *ap^DG1^*. Therefore, homo- or hemi-zygous *ap^attPΔEnh^* flies have no wings (data not shown). The attP site of *ap^attPΔEnh^* serves as docking site for *ΦC31*-mediated integration of any desired DNA located on a plasmid containing an attB site and the *yellow* selection marker (Figure 5, D and E).

**Figure 6:**
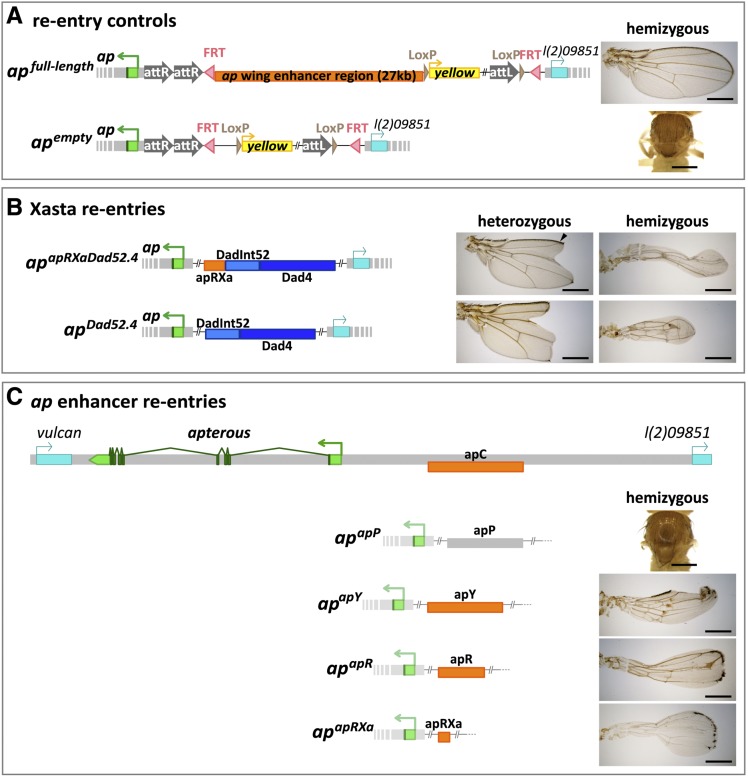
Testing *dad* and *ap* enhancers in the endogenous *apterous* locus. (A) Positive control: the whole 27-kb *ap* wing enhancer region was re-inserted and *ap^full-length^* flies obtained. In *ap^full-length^*/*ap^DG3^* animals, perfectly wild-type wings are formed. Negative control: the empty pEnh-Reentry plasmid gave rise to *ap^empty^* flies. No wing tissue is formed in *ap^empty^*/*ap^DG3^* adults. (B) *Xasta* phenocopies are obtained with *ap^apRXaDad52.4^* and *ap^Dad52.4^* alleles. *ap^apRXaDad52.4^* contains three enhancer elements: apRXa, DadInt52, and Dad4. Heterozygous flies only produce rather weak phenocopies. The junction between wing vein L2 and the margin (see arrow head) is present in almost 100% of the wings. *ap^Dad52.4^* contains only the 2 *dad* enhancers. Faithful phenocopies of *ap^Xa^/+* wings where the junction between vein L2 and the margin is missing are often observed. The wing phenotypes of hemizygous *ap^apRXaDad52.4^*, *ap^Dad52.4^* and *ap^Xa^* are similar: tube-like wing stumps of variable length are formed. Margin bristles are absent except for sometimes a few at the tip of the wing. (C) Testing the wing enhancer activity of four apC derivatives. At the top of the panel, the *ap* locus is depicted. Below, the positions of fragments apP, apY, apR and apRXa are shown relative to apC. The respective wing phenotypes in hemizygous condition are shown to the right of the corresponding fragments. Flies transgenic for the gray apP fragment behave like a true null allele: no wings are formed. Fragments drawn in orange have partial rescue activity: inflated wings are formed, where most of the margin and the alula are missing. The hinge is poorly formed. Note that in B and C, for space reasons, parts of the reentry plasmid have been omitted. All scale bars are 50 µm.

As proof of principle, two control plasmids were first introduced into *ap^attPΔEnh^*: (1) the empty pEnh-Reentry vector gave rise to a fly line called *ap^empty^*; (2) pEnh-Reentry-full-length contained the complete 27-kb intergenic spacer and the corresponding transgenic line was called *ap^full-length^* (Figure 6A). The “wing-forming” activity of these two controls as well as all subsequent transgenic lines analyzed in this study was determined in hemizygous condition. Therefore, balanced *ap^empty^* and *ap^full-length^* males were crossed with *ap^DG3^/SM6* virgins and the wings of *trans*-heterozygous progeny were carefully inspected. As expected, *ap^empty^/ap^DG3^* flies generated no detectable wing material. In contrast, the reconstituted *ap* locus produced wild-type wings in *ap^full-length^/ap^DG3^* flies. Taken together, these observations demonstrate the feasibility of our *in situ* rescue system and suggest that the backbone of the pEnh-Reentry plasmid does not cause any disturbances.

### DadInt52 and Dad4 enhancers contribute significantly to the Xasta phenotype

Our model for the Xasta wing phenotype posits that the wing specific *dad* enhancers Dad4 and DadInt52 are responsible for ectopic Ap expression in the ventral pouch compartment. We wished to test this hypothesis with the *in situ* rescue system. Two fly lines were established: *ap^apRXaDad52.4^* and *ap^Dad52.4^* (Figure 6B). The former combined the three identified wing specific enhancers apRXa, DadInt52 and Dad4. The latter contained only the two *dad* regulatory elements. When the 2 transgenics were initially isolated, it was immediately apparent that both phenocopied the dominant *Xasta* allele. However, a semi quantitative analysis also showed that the severity of their phenotypes was weaker than observed for *ap^Xa^/+* wings (Table 2). Although roughly 50% of the *ap^Dad52.4^/+* wings were as strongly affected as those of *ap^Xa^/+* flies, hardly any such wings appeared in *ap^apRXaDad52.4^/+* flies. These observations indicate that apart from DadInt52 and Dad4, other factors contribute to the production of a full blown Xasta wing phenotype.

**Table 2:** Penetrance of the dominant *ap^X^**^a^* wing phenotype

Genotype	Number of Wings Scored*^a^*	L2 Junction Present	L2 Junction Absent
*ap^X^**^a^**/+*	262	6.5%	93.5%
*ap^apRXaDad52.4^/+*	160	98.1%	1.9%
*ap^Dad52.4^/+*	546	58.6%	41.4%

a Wings were scored for the presence or absence of the junction between wing vein L2 and the wing margin.

The wings of *ap^apRXaDad52.4^* and *ap^Dad52.4^* were also analyzed in hemizygous condition. The phenotypes were comparable to the one seen in *ap^Xa^*/ *ap^DG3^* flies: only tube-like wing stumps were formed which lacked wing margin completely except for the occasional occurrence of a few margin hairs at the very tip. It is conceivable that the latter arise due to the Wg spot seen in the center of the pouch of *ap^Xa^/ap^DG3^* wing discs (see Figure 3B). We have never seen homozygous *ap^Xa^* flies but did inspect adult wings of *ap^Xa^/ap^Dad52.4^* animals. They appeared as even smaller versions of those observed in hemizygous *ap^Xa^* flies (data not shown).

### The apRXA enhancer is required but not sufficient for wing formation

In Figure 1 of this paper, we have presented evidence that the ∼8 kb apC fragment harbors an 874-bp wing specific enhancer that is essential for wing formation. However, the experimental approaches we used are not adequate to test whether the enhancer is also sufficient for the formation of a wild-type wing. Therefore, four overlapping fragments covering the whole apC were introduced into the *ap* locus and the corresponding transgenic lines were obtained: *ap^apP^*, *ap^apY^*, *ap^apR^*, and *ap^apRXa^*. Their wing enhancer activity was tested in a hemizygous genetic background (Figure 6C). *ap^apP^/ap^DG3^* flies, which contained the apP fragment that did not yield any LacZ reporter activity (see Figure 1A), also did not develop any wing or haltere tissue and phenotypically resembled *ap* null alleles. When apY, a fragment which is shifted by 2 kb toward the *ap* TSS, was tested in *ap^apY^/ap^DG3^* flies, wing development was partially restored. However, most of the margin, the alula and the hinge region were poorly formed. Similar phenotypes as for *ap^apY^* were observed in *ap^apR^/ap^DG3^* and *ap^apRXa^/ap^DG3^* flies. Note that these three apC derivatives were sufficient to drive *ap*-specific *LacZ* expression in our reporter assay (see Figure 1A). “Homozygotes” obtained by pairwise combinations of *ap^apY^*, *ap^apR^* or *ap^apRXa^* were also studied. Such wings looked improved compared to the phenotypes observed in hemizygotes, because the margins, particularly along the anterior but also along the posterior edges of the wing, were formed to a large degree (data not shown). Somewhat unexpectedly, heterozygous *ap^apY^*, *ap^apR^* and *ap^apRXa^* flies showed a weak dominant wing phenotype, associated with a small notch in the tip region in 10–20% of the cases. This phenotype was not observed in *ap^apY^*/+ or *ap^full-length^/ap^DG3^* flies (data not shown).

These results demonstrate that the 874 bp apRXa wing enhancer element is required but not sufficient in the endogenous context to correctly regulate *ap* expression. Our observations imply the existence of further unidentified wing enhancer elements elsewhere in the *ap* region.

## Discussion

In the past, *cis*-regulatory elements were mainly investigated using reporter-based assay systems, in which putative regulatory DNA fragments were tested for their ability to drive reporter gene expression when present on a transgene inserted randomly in the genome (Simon *et al.* 1985; Hiromi and Gehring 1987). Although this method proved to be a highly useful and valuable approach, it has some shortcomings. Enhancer fragments are tested in a genomic environment that may differ considerably from their native position. Additionally, the results of such studies yield little or no information about whether the investigated elements are sufficient, permissive or even dispensable for the regulation of gene expression at their original location. Recently, some improvements were achieved by using bacterial artificial chromosomes to investigate *cis*-regulatory elements in a broader genomic context (Dunipace *et al.* 2013).

To circumvent the problem of positional effects, we performed our classical reporter assay at a single *ΦC31*-system docking site located on 3R. Our laboratory has successfully used this insertion site for the analysis of wing specific enhancer elements (Weiss *et al.* 2010). Furthermore, we investigated the relevance of the reporter data with two powerful genetic approaches. We used methods from the *Drosophila* genetic tool kit and generated useful materials for the *in situ* dissection of regulatory elements directly at the *ap* locus. First, a set of small overlapping deletions within the *ap* region was isolated with the help of different transposable elements carrying FRT sites. Second, the *in situ* rescue system was established. The novel fly strain generated greatly facilitates the introduction of any DNA fragment by means of integrase-mediated recombination into the *apterous* locus. It has and will serve us as a tool to dissect important *ap* regulatory sequences in great detail.

In this study, the combined application of reporter assay, deletion analysis and *in situ* rescue system has allowed us to firmly establish the 874 bp apRXa fragment as an essential wing-specific regulatory element for *apterous* transcription. We show that apRXa is sufficient to drive reporter gene expression within the dorsal compartment of the wing pouch. Flies hemizygous for an 11-kb deletion encompassing the apRXa element develop no wing structures. This observation proves that this larger DNA interval including apRXa is essential for *ap* function. Finally, when tested in the context of the endogenous *ap* locus, we document that apRXa is required but not sufficient to form wild-type wings.

The importance of the apRXa enhancer element is further highlighted through the molecular characterization of 2 classical *ap* alleles, *ap^blot^* and *ap^Xa^*. *ap^blot^* contains an insertion of a retrotransposon from the *blood* family. This insertion is located within the apRXa enhancer. We have not attempted to prove the presence of the full length 7.4-kb *blood* element in *ap^blot^*, but we have completely sequenced both LTRs. So far, all *blood* elements detected in the *Drosophila* genome are full-length insertions. None of them was found to be truncated (Kaminker *et al.* 2002). Hence, it appears likely that *ap^blot^* also contains an intact, full length *blood* element and that it is accountable for the mutagenic effect. For example, it is possible that the insertion destroys an important transcription factor binding site within the apRXa wing enhancer. Alternatively, the inserted DNA might separate important transcription factor binding sites.

The other *ap* allele we investigated is *ap^Xa^*. In this mutant, a reciprocal translocation event between the right arms of the second and third chromosomes caused a breakpoint immediately upstream of the apRXa wing enhancer. This rearrangement juxtaposes the *dad* locus next to *apterous*. Our experimental evidence strongly indicates that in this mutant, *ap* transcription falls under the control of *dad* wing specific enhancers Dad4 and DadInt52. As a consequence, *ap* and its target genes are ectopically expressed in the medial section of the ventral part of the wing disc, conferring ventral cells with a dorsal cell fate identity. This, in turn, likely interferes with signaling at the D-V compartment boundary and causes the disruption of the Wg stripe in the center of the wing pouch. *dad*-controlled *ap* expression also provides an explanation why the anterior compartment is more strongly affected than the posterior one in adult wings. As evidenced by asymmetric Dad4-GFP expression along the A-P compartment boundary, a wider domain with higher levels of GFP is produced in the anterior compartment (see Figure 3C). We propose that a similar asymmetrical distribution of Ap causes differential Wg stripe expression in the two compartments.

Our observations also suggest that *dad*-mediated transcriptional activation of *ap* is not the sole cause for the explanation of the Xasta phenotype. The dominant phenotypes of *ap^apRXaDad52.4^/+* and *ap^Dad52.4^/+* flies are clearly less pronounced than that documented for *ap^Xa^/+*. Why should this be the case? It is known that the *apterous* locus is a target of the repressive Polycomb Group (PcG) system. *Scm****^−/−^*** clones reaching into the ventral compartment elicit ectopic Ap expression (Oktaba *et al.* 2008). In addition, it is well documented that the silencing activity of isolated Polycomb Response Elements is pairing dependent (Kassis *et al.* 1991; Fauvarque and Dura 1993; Chan *et al.* 1994; Gindhart and Kaufman 1995; Muller *et al.* 1999; reviewed in Kassis 2002). It is therefore conceivable that in *ap^Xa^/+* flies, the chromosomal rearrangement prevents efficient homologous chromosome pairing and thus reduced PcG-mediated silencing. This hypothesis is supported by the fact that the *mini-white* markers of transgenes inserted in the *ap* locus are partially derepressed in a *Xasta* heterozygous background (M. Müller, unpublished data). An alternative explanation could be that as yet-uncharacterized wing-specific enhancers are present in the genomic *dad* locus which lack in *ap^apRXaDad52.4^/+* and *ap^Dad52.4^/+* flies. It thus might be that the stronger phenotype observed in *ap^Xa^*/+ flies is a consequence of stronger Ap misexpression due to the combined effect of more than two enhancers.

Our findings imply the existence of other, as yet unidentified wing-specific regulatory elements within the realms of the *apterous* locus. A hint about the possible location of such sequences has previously been obtained through the genetic analysis of insertion *PBac{WH}f00451*. This transposon is located about 3 kb distal to apRXa. The PBac{WH} element contains an array of Su(Hw) binding sites at its 3′ end (Thibault *et al.* 2004). It is well established that on transgenes, a cluster of Su(Hw) binding sites acts as enhancer blocker. It interferes with enhancer-promoter interaction when placed in between two such regulatory elements (Holdridge and Dorsett 1991; Hagstrom *et al.* 1996; Scott *et al.* 1999). Furthermore, the mutagenic effect of an array of Su(Hw) binding sites located on the *gypsy* mobile genetic element can often be attributed to a similar mechanism (Geyer *et al.* 1986; Peifer and Bender 1988; Dorsett 1993; Hogga *et al.* 2001). Homozygous as well as hemizygous *ap^f00451^* flies have been reported to cause a rather strong wing phenotype. Importantly, the phenotype is completely suppressed in a *su(Hw)*^-^ background (Gohl *et al.* 2008). This observation suggests the presence of other *ap* wing enhancer elements distally to *PBac{WH}f00451*. We are currently exploring this possibility with analogous experimental approaches as outlined above, in particular through the use of the *in situ* rescue system established and described in this study (D. Bieli *et al.*, unpublished results).

## Supplementary Material

Supporting Information
