# Supplementary material for: The Drosophila melanogaster Mutants apblot and apXasta Affect an Essential apterous Wing Enhancer
Source: G3 (Bethesda). 2015 Apr 2;5(6):1129–43. doi: 10.1534/g3.115.017707 (PMC4478543; doi:10.1534/g3.115.017707)
Supplement: Supporting Information [file supp_5_6_1129__index.html]

The Drosophila melanogaster Mutants apblot and apXasta Affect an Essential apterous Wing Enhancer — Supporting Information 

# The *Drosophila melanogaster* Mutants *apblot* and *apXasta* Affect an Essential *apterous* Wing Enhancer

## Supporting Information for Bieli *et al.*, 2015

**Files in this Data Supplement:**

- Table S1 - Primers (.xlsx, 12 KB)
